# Comparison between Two Molecular Techniques: Nested and Real-Time Polymerase Chain Reaction Targeting 100-kDa Hc Protein for Detection of *Histoplasma capsulatum* in Environmental Samples

**DOI:** 10.4269/ajtmh.20-1396

**Published:** 2022-03-14

**Authors:** Luisa F. Gómez, Lalitha Gade, Anastasia P. Litvintseva, Juan G. McEwen, Carlos A. Peláez, Myrtha Arango, María del P. Jiménez

**Affiliations:** ^1^ Medical Mycology Group, School of Medicine, Microbiology and Parasitology Department, Universidad de Antioquia, Medellin, Antioquia, Colombia;; ^2^ Biology Postgrad Program, Institute of Biology, School of Exact and Natural Sciences, Universidad de Antioquia, Medellín, Antioquia, Colombia;; ^3^ Mycotic Diseases Branch, Centers for Disease Control and Prevention, Atlanta, Georgia;; ^4^ Cellular and Molecular Biology Group, Corporación para Investigaciones Biológicas (CIB), Universidad de Antioquia, Medellín, Antioquia, Colombia;; ^5^ Grupo Interdisciplinario de Estudios Moleculares, Institute of Chemistry, School of Exact and Natural Sciences, Universidad de Antioquia, Medellín, Antioquia, Colombia

## Abstract

Histoplasmosis, one of the most frequent endemic mycoses in the Americas, is caused by the inhalation of airborne conidia of *Histoplasma capsulatum*. Better understanding of the distribution of this fungus in the environment is important for the development of appropriate public health measures to prevent human infections. Previously, we used Hc100 nested polymerase chain reaction (PCR) to identify *H. capsulatum* DNA in 10% of environmental samples in Colombia. Here, we validate a 100-kDa real-time PCR assay for the detection of this fungus in the environment. Using this method, we identified *H. capsulatum* DNA in 80% of samples of raw organic materials, such as chicken manure, soil from caves, and bird and bat guano, as well as in 62% of samples of organic fertilizer that underwent the composting process. We demonstrated that 100-KDa real-time PCR is a useful tool for environmental surveillance that can be used to identify the potential reservoirs of *H. capsulatum* and to prevent outbreaks, especially in people with the higher risk of exposure, such as spelunkers, farmers, poultry manure collectors, and anyone who handle organic fertilizers or bat and bird excreta.

## INTRODUCTION

Histoplasmosis is reported around the world and is especially widespread in the Americas, where it is considered one of the most frequent endemic mycoses. The infection is caused by the thermally dimorphic fungus *Histoplasma capsulatum.* Infections occur in both immunocompromised and immunocompetent populations with a vast spectrum of clinical presentations from asymptomatic to disseminated lethal disease.^1^ Better understanding of the distribution of this fungus in the environment is essential for delimiting the potential sources of histoplasmosis to protect especially vulnerable populations, such as patients with HIV/AIDS, organ transplants, and other immunosuppressing conditions.^1–4^ Until recently, most environmental surveillance efforts have been focused on outbreak investigations.^3,5–7^ For example, in the 2001 outbreak of histoplasmosis in a hotel in Acapulco, Mexico, which affected more than 700 people, the infection was linked to plants that were located near the restaurant entrance and recently fertilized with a contaminated chicken manure.^5,6^ Similarly, a smaller outbreak in Colombia involved a family infected with histoplasmosis after handling contaminated organic fertilizer.^3^ Broader environmental investigations are hampered by the inefficacy of the current detection methods, which still rely on costly and inefficient methods such as animal inoculations and culture.

The first published attempt to identify *H. capsulatum* using molecular methods was conducted in 1999 by Reid and Schafer, who designed a nested polymerase chain reaction (PCR) assay targeting the internal transcribed spacer (ITS) of the 5.8S ribosomal RNA (rRNA); however, this method only worked on pure cultures of *H. capsulatum*.^8^ In 2001, the Hc100 nested PCR technique was developed by Bialek et al., and since then, it has been widely used for diagnosis in human clinical samples because of its high sensitivity and specificity and its easy implementation into laboratory workflows.^9–12^ In 2012, Frias de Leon et al. developed two PCR assays based on sequence-characterized amplified region (SCAR) markers of *H. capsulatum*, generated by randomly amplified polymorphic DNA (RAPD)-PCR that enabled the detection of the fungus DNA in clinical and environmental samples.^13^ Lopez et al. designed a real-time PCR for the detection of *H. capsulatum* DNA in clinical samples that targeted the same region of 100-kDa protein as the Hc100 nested PCR assay but had better sensitivity and specificity than the nested PCR assay.^14^

Previously, our team used the Hc100 nested PCR for environmental testing and demonstrated the presence of *H. capsulatum* DNA in 10% of tested soil and organic fertilizers sampled in Colombia. Notably, the majority of organic fertilizers used in that study were approved in accordance with the Colombian Technical Standard (Norma Técnica Colombiana) 5167, which regulates the microbiological, physical, and chemical characteristics of organic fertilizers for commercial use,^15,16^ yet they contained evidence of *H. capsulatum* contamination. In this current study, we validate the 100-KDa real-time PCR assay for environmental detection and use this highly sensitive and specific method to further investigate the prevalence of *H. capsulatum* in the environment and in organic fertilizers in Colombia.

## MATERIALS AND METHODS

### DNA from environmental samples.

A subset of 332 of organic fertilizer, soils from cave floors, and bat droppings from the previous study collected in 2010–2014^15^ were included in this investigation. These samples were sent to the Interdisciplinary Group of Molecular Studies at Chemistry Institute, Universidad de Antioquia, by producers of organic fertilizer from different places of the Andean region of Colombia.

Total DNA was extracted using the FastDNA SPIN Kit For Soil (MP Biomedicals, Santa Ana, CA) following the manufacturer’s instructions with some modifications. Briefly, the extraction was performed using 300 µL of the supernatant obtained from the suspension of 10 g of sample in 30 mL of a supplemented saline solution. During the study mentioned earlier, the DNA samples were declared free of PCR inhibitors.^15^

### Hc100 nested PCR.

The Hc100 nested PCR assay in environmental samples was performed as described by Gomez et al.^15^ The PCR assay was done with two sets of specific primers for *H. capsulatum*, the external pair HcI and HcII and the internal pair HcIII and HcIV. Primers were previously designed by Bialek et al.^10^ The target of these primers is a fragment of the sequence of the single copy gene encoding a 100-kDa protein that is constitutive in the *H. capsulatum* genome.^17^ The external primers amplified a 391-bp fragment within which the internal primers annealed and amplified a 210 bp fragment. The presence of this amplification product was considered positive for *H. capsulatum*.

### 100-kDa real-time PCR assay.

Lopez et al. published the design, standardization, and validation of three real-time PCR assays for detecting *H*. *capsulatum* DNA in fresh and formalin-fixed paraffin-embedded (FFPE) tissues. According to their results, the real-time PCR assay targeting the 100-kDa protein gene showed 100% specificity and analytical sensitivity, with a detection limit of two DNA copies. This assay was selected to detect DNA of *H. capsulatum* in the environmental samples.^14^

The real-time PCR conditions were as follow: a mixture (25 µL) that contained 1X Maxima Probe qPCR Master Mix (2X) (Thermo Fisher Scientific, Waltham, MA), 0.2-μM primers 84R22 (forward) and 15F23 (reverse), 0.3-μM probe 45L23, and 1 ng genomic DNA. Real-Time PCR was performed using a BioRad thermocycler (BioRad, Hercules, CA) under the following conditions: 95°C for 10 minutes, followed for 50 cycles of 95°C for 15 seconds, 58°C for 30 seconds, and 72°C for 10 seconds. The amplicons generated had lengths of 90 bp.

### Determination of detection sensitivity and specificity of the 100-kDa real-time PCR assay.

The sensitivity to detect DNA of *H. capsulatum* in the environmental samples was calculated based on the comparison of the results by the real-time PCR assay with the culture and the previously performed Hc100 nested PCR assay. Positive-control samples were environmental samples that had been demonstrated positive for *H. capsulatum* by Hc100 nested PCR assay (*n* = 38) and microbiological culture (*n* = 1).^18,19^ Detection specificity was calculated by using 11 negative-control soils samples from nonendemic areas. To test for the possibility of cross-contamination during sample handling, we tested a sterile distilled-water sample that was processed along with the environmental samples and subjected to the same DNA extraction procedures.

### Search for *H. capsulatum* in organic fertilizers by 100-kDa real-time PCR assay.

DNA from 332 environmental samples was evaluated in triplicate for the detection of *H. capsulatum*. Each round of real-time PCR testing included a positive control consisting of DNA purified from a culture of *H. capsulatum* in the yeast phase (1 ng/µL) and sterile distilled water sample, as a negative control.

### Purification and sequencing of the products amplified by 100-kDa real-time PCR.

From the amplified real-time PCR products, 5% (*n*= 15) were purified using ExoSAP (Affymetrix, Santa Clara, CA) according to the manufacturer’s instructions. Cycle sequencing reactions (20 μL) were prepared with 2 µL of DNA template and 3.2 pmol of the same primers used for PCR, 2 µL of BigDye Terminator v3.1, 2 µL of sequencing buffer (Applied Biosystems, Inc., Waltham, MA) according to the manufacturer’s instructions. Extension products were purified using Centri-Sep plates (Princeton Separations, Inc., Freehold, NJ) and electrophoresed on a 3730 DNA analyzer (Applied Biosystems, Inc.). The obtained sequences were edited manually based on the chromatograms, and the mold and complementary sequences were identified using Geneious 11.0.2. Software. BLAST (http://blast.ncbi.nlm.nih.gov/Blast.cgi) was used to verify that the sequenced PCR products belonged to *H. capsulatum.*

## RESULTS

### Concordance between 100-kDa real-time and Hc100 nested PCR assays.

The performance of 100-kDa real-time PCR was first tested on a subset of 49 well-characterized environmental samples from the previous study that included: 39 positive samples and 10 negative samples by Hc100 nested PCR. Of those, all samples positive for *H. capsulatum* by the Hc100 nested PCR assay also were found positive by the real-time PCR assay. The 10 samples from nonendemic areas that tested negative in the previous study were also found negative by the new assay, demonstrating concordance between the two methods. Real-time PCR products from 15 positive samples were sequenced and showed 100% identity with the corresponding fragment of HC100 gene of *H. capsulatum*, confirming the specificity of this assay (data not shown).

### Detection of *H. capsulatum* DNA in environmental samples.

We then tested 332 environmental samples from various environmental sources in Colombia by two PCR assays. *H. capsulatum* DNA was detected in 223 of these samples by 100-kDa real-time PCR (67% positivity rate) and in 38 samples by Hc100 nested PCR (11% positivity rate; Table 1). However, all 38 samples positive by Hc100 nested PCR were also positive by 100-kDa real-time PCR. The median cycle threshold (Ct) value for samples positive by the both assays was 34.3, and the median Ct value for samples positive by the real-time PCR assay was 34.9; however, the difference between the Ct values for these two groups was not statistically significant (Mann-Whitney test, *P* = 0.23)

**Table 1:** Results of the environmental samples obtained by Hc100 nested polymerase chain reaction (PCR) and 100-kDa real-time PCR

		No. positives (%) by				
			Ct values	
Results	No. of samples tested	Hc100 nested PCR	%	100 KDa real-time PCR	Range (median)	%
All samples	332	38	11	223	23.9–39.4 (34.8)	67
Composted organic fertilizers	241	21	9	150	25.7–39.4 (34.9)	62
Raw samples	91	17	19	73	23.9–38.3 (34.5)	80
Chicken manure	75	16	21	60	23.9–38.3 (34.5)	80
Caves soils or bat droppings	16	1	6	13	33.6–36.9 (34.9)	81

Ct = cycle threshold.

Of the 332 samples, 91 were from natural sources that did not undergo composting, including raw chicken manure (*n* = 75) and soils from cave floors or bat droppings (*n *= 16, Table 1). Of those, 73 (80%) were found to contain *H. capsulatum* DNA by 100-kDa real-time PCR, and 17 (27%) were found positive by Hc100 nested PCR (Table 1). The remaining 241 samples were composted organic fertilizers; of those, 150 (62%) were found to contain *H. capsulatum* DNA by 100-kDa real-time PCR, and 21(9%) were found positive Hc100 nested PCR (Table 1). The difference in the proportions of positive samples between composted and noncomposted sources was not statistically significant (Fisher’s exact test, *P* = 0.18). The median Ct value for positive composted samples was 34. 9, and the median Ct value for positive raw samples was 34.5; however, this difference in Ct values between the two groups was not statistically significant (Mann-Whitney test, *P* = 0.9)

## DISCUSSION

We evaluated the performances of 100-kDa real-time and Hc100 nested PCR for the detection of *H. capsulatum* DNA in environmental samples and used these assays to investigate the presence of this fungus in these substrates in Colombia. Our results demonstrated that although both assays considerably outperformed culture, which was only able to detect *H. capsulatum* in a single sample,^18,19^ 100-kDa real-time PCR was able to identify 600% more positive samples compared with the Hc100 nested PCR assay. The higher sensitivity of real-time PCR compared with nested PCR was not surprising, given that the real-time PCR assay did not rely on visualization of PCR products on the agarose gel, and therefore, lower concentrations of PCR products could be detected with this method. Similar results were obtained in other studies comparing the performances of the real-rime and conventional PCR assays.^20^ The specificity of 100-kDa real-time PCR was validated by sequencing amplification products from a subset of positive reactions, which demonstrated identity of the real-time PCR product with the corresponding region of the *H. capsulatum* Hc100 gene and increased confidence in the molecular detection results.

Our results showed that 80% of samples from natural sources included in our study contained *H. capsulatum* DNA detectable by 100-kDa real-time PCR. Such high positivity among these samples was not surprising considering the high prevalence of histoplasmosis in Colombia and the fact that these samples were collected from typical *H. capsulatum* habitats, such as bird and bat excreta, soil from cave floors, and poultry houses in Colombia.^7,15,21–23^ In addition, to our surprise, 62% of organic fertilizers that underwent composting before testing were also found to contain *H. capsulatum* DNA by real-time PCR. Although the percentage of positive samples among composted samples was lower than that observed among the natural noncomposted samples, the difference between two types of samples was not statistically significant, and the observed positivity rate among composted samples was still notably high.

Our previous studies demonstrated that properly performed composting can decrease the amount of detectable *H. capsulatum* in organic fertilizers.^19^ Several factors may explain the observed high proportion of positive samples among composted fertilizers in this study. First, it is possible that the highly sensitive 100-kDa real-time PCR assay detects DNA from nonviable fungus, and the positive signal indicates the history of *H. capsulatum* contamination in the composted fertilizers. Second, it is also possible that the length or efficiency of composting was not enough to eliminate all *H. capsulatum* from the fertilizers and the pathogen can be detected with this more superior molecular method. Although, to our knowledge, no data exist on the stability of DNA in soil or compost, it is unlikely that free DNA from the nonviable cells would persist in samples with high microbial load. Finally, it is also possible that the composted fertilizers became contaminated again during storage or transportation process. This last possibility is especially plausible considering the overall high prevalence of this pathogen in the Colombian environment. More research is needed to investigate the viability and infectivity of *H. capsulatum* in composted materials. Importantly, our results indicate that *H. capsulatum* is frequently present in raw materials used for production of organic fertilizers and its DNA can be detected in composted products; therefore, proper treatment and quality control measures should be considered to ensure safety of these products.

The detection of *H. capsulatum* DNA by the 100-kDa real-time PCR in the 67% of the overall samples indicates presence of the fungus in the Colombian soil. These results, combined with data from epidemiological surveillance, suggest that the reported incidence of histoplasmosis may be an underestimate. Knowledge of environmental foci could be improved by public health reporting of the disease and the implementation of an environmental surveillance system. Molecular detection methods described in our study provide important tools for environmental surveillance that can lead to the development of the preventive measures to reduce the occurrence of outbreaks and cases of histoplasmosis among farmers, gardeners, poultry manure collectors, and any who handle organic fertilizers or bat and chicken excreta.
